# Evaluation of the activity of CYP2C19 in Gujrati and Marwadi subjects living in Mumbai (Bombay)

**DOI:** 10.1186/1472-6904-6-8

**Published:** 2006-10-24

**Authors:** Tanmay S Panchabhai, Shaun F Noronha, Sanish Davis, Vishal M Shinde, Nilima A Kshirsagar, Nithya J Gogtay

**Affiliations:** 1 Department of Clinical Pharmacology, Seth GS Medical College and KEM Hospital, Parel, Mumbai 400012, India

## Abstract

**Background:**

Inherited differences in the metabolism and disposition of drugs, and genetic polymorphisms in the targets of drug therapy (e.g., receptors), can greatly influence efficacy and toxicity of medications. Marked interethnic differences in CYP2C19 (a member of the cytochrome P-450 enzyme superfamily catalyzing phase I drug metabolism) which affects the metabolism of a number of clinically important drugs have been documented. The present study evaluated the activity of CYP2C19 in normal, healthy Gujrati and Marwadi subjects by phenotyping (a western Indian population).

**Methods:**

All subjects received 20 mg of omeprazole, which was followed by blood collection at 3 hrs to estimate the metabolic ratio of omeprazole to 5-hydroxyomeprazole. The analysis was done by HPLC.

**Results:**

It was seen that 10.36% of this population were poor metabolizers(PM) whereas 89.63% were extensive metabolizers(EM).

**Conclusion:**

A genotyping evaluation would better help in identifying population specific genotypes and thus help individualize drug therapy.

## Background

The differences among individuals in the way they respond to medications [1] can be attributed to inherited differences in the metabolism and disposition of drugs, and genetic polymorphisms in the targets of drug therapy (e.g., receptors) [2-4] other than the conventional factors like individual's age and race, organ function, concomitant therapy, drug interactions, and concomitant illnesses [5]. CYP2C19, a member of the cytochrome P-450 enzyme superfamily(catalyzing phase I drug metabolism) [6] affects the metabolism of a number of clinically important drugs, such as proton pump inhibitors (omeprazole [7], lanzoprazole, rabeprazole), tricyclic antidepressants (imipramine, amitryptiline), phenytoin, propranolol and benzodiazepines (diazepam) [8]. Marked interethnic differences in the polymorphism frequency [6,9] have led to 21 variant alleles (CYP2C19*2 to CYP2C19*8) being documented; that predict poor metabolizers (PMs); of which the most commonly encountered ones contributing to a PM phenotype were CYP2C19*2 and CYP2C19*3 genotypes. The prevalence of PMs has been reported to be 2–5% in Caucasians [10,11], 4–8 % in Africans [12] and 11–23 % in Orientals [11].

Polymorphisms can be determined by phenotyping and genotyping methodology. The phenotyping method employs the use of "Probe Drugs". These are drugs which are characteristically metabolized by a single enzyme system and hence can be used to classify individuals as extensive metabolizers (EMs) or poor metabolizers (PMs). The disadvantages in using older probe drugs like mephenytoin has prompted the use of others like omeprazole [13] and proguanil [14]. The fact that omeprazole is almost exclusively metabolized by CYP2C19 to 5-hydroxy omeprazole and to a lesser extent by CYP3A4 to omeprazole sulphone makes it a valuable probe drug for establishing the genotype-phenotype correlation for CYP2C19.

Omeprazole is used in combination regimens for the treatment of gastric as well as duodenal ulcers, gastroesophageal reflux disease and for eradication of *Helicobacter pylori *infection. There exist significant differences in intragastric pH [15] and differences in cure rates for *H. pylori *infection [16] between extensive metabolizers (EMs) and poor metabolizers (PMs) who are treated using omeprazole. Gastric acid suppression and eradication of *H. pylori *infection are important determinants in the management of the pathologies mentioned *vide supra*. It has been shown that a higher concentration of omeprazole in PMs results in greater gastric acid suppression as compared with extensive metabolizers [15]. Whereas the frequency of these polymorphisms in North [17] and South [18] Indians (who respectively belong to Aryan and Dravidian races) has been documented, the variations in CYP2C19 activity in Western Indian population has not been determined so far. The present study thus evaluated the activity of CYP2C19 in normal, healthy, Gujrati and Marwadi subjects by phenotyping using omeprazole as the probe drug.

## Methods

### Ethics

The study was conducted after approval from the Institutional Review Board, and in accordance with Ethical Guidelines for Biomedical Research in Human subjects of ICMR (2000) [19]. Written, informed consent was obtained from all participating subjects.

### Study procedure

The study was conducted in 170 normal healthy (by history and focused clinical examination) Gujrati and Marwadi subjects residing in the state of Maharashtra, ensuring that their native places were in the states of Gujrat and Rajasthan. The sample size was calculated assuming a 12% prevalence of PMs with 95% CI at 5% significance. The prevalence for the sample size calculation was taken as 12% based on the data of 14% and 12% prevalence in North [14] and South [15] India respectively. Subjects were admitted to the Clinical Pharmacology ward and received Omeprazole 20 mg (Lomac-20^®^, batch no: G57688, Cipla Ltd, Mumbai, India) after an overnight fast. The drug was administered orally under direct supervision and the blood sample (10 ml of venous blood collected from an antecubital vein into 1 tube containing 100 μL Heparin) was collected in accordance with the protocol 3 hours after ingestion of the drug. Alcohol, caffeine and citrus juices were avoided for at least 48 hours before the intake of omeprazole. The plasma was separated and stored at -20°C pending further analysis. Omeprazole and 5-hydroxy omeprazole plasma concentrations were determined by reverse phase high performance liquid chromatography. The analytes were detected at 302 nm and absorbance was set at 0.005 Aufs. The sensitivity of the method and percentage extraction was 10 ng/ml and 90.0% for both drug and metabolite. The assay was found to be linear over the concentration range from 0.1 to 10.00 μg/ml with all the concentrations giving precision and accuracy within 15%. The phenotyping was done based on minor modifications of the method of Kobayashi et al. [20]. Statistical analysis was performed using the Graphpad Instat statistical software (Graph Pad Software Inc., San Diego, CA, USA). The Kolmogorov-Smirnov test was used to test normality in the metabolic ratios.

## Results

Of 170 samples 6 samples could not be analyzed for various reasons and phenotyping was done for the remaining 164 samples. There were 110 males (67.07%) and 54 females (32.92%), the mean age of the population being 24.79 ± 11.51 (mean ± SD)(95%CI; 26.55, 23.03). Of the 164 subjects, there were 122 Gujratis (77 males and 45 females) and 42 Marwadis (32 males and 10 females). No subject reported any adverse event to the single dose of the drug.

**Figure 1:**
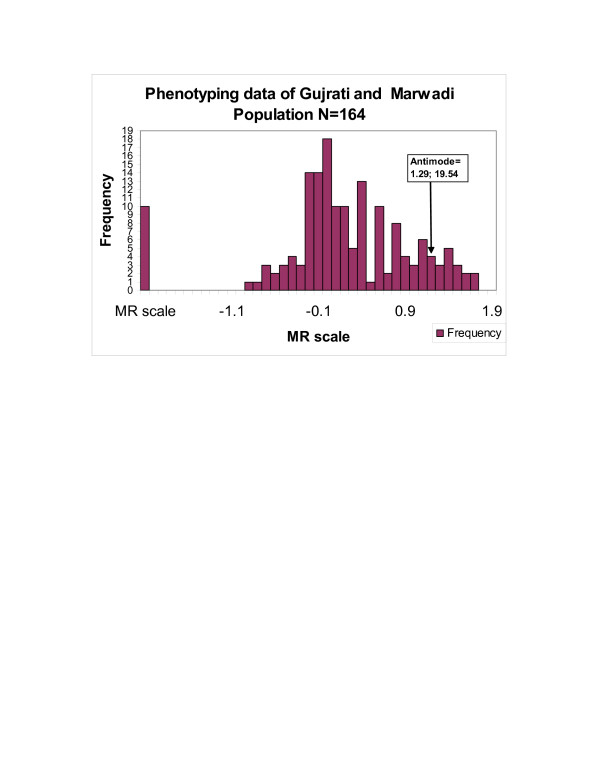
Frequency distribution curve of the phenotyping data showing demarcation of extensive (EMs) and poor metabolizers (PMs) based on antimode calculations. Those with antimode greater than 19.54 classified as PMs.

**Figure 2:**
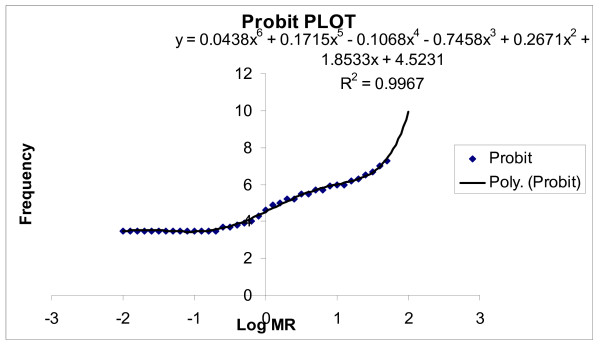
Probit plot obtained by curve fitting the log MR ratio data.

The frequency histogram (Figure 1) and the probit plot (figure 2) of 164 subjects confirmed a bimodal distribution of subjects with respect to their Metabolic Ratio (MR). The antimode in the Gujrati and Marwadi (Western Indian) population was calculated to be 19.54 (log MR = 1.291). Individuals with an MR >19.54 were categorized as poor metabolizers (PMs), whereas those with MR<19.54 were categorized as extensive metabolizers (EMs). The mean MR of the population was 6.52 ± 0.85 (mean ± SEM) (95%CI; 8.198,4.846).

In the study group of 164 volunteers,147 (89.63%) were EMs with a mean MR of 3.27 ± 0.36 (mean ± SEM) and 17 (10.36%) were PMs with a mean MR of 36.85 ± 3.32 (mean ± SEM). It was further observed that the 55 females, 5(09.09%) were PMs and 50(91.91%) were EMs. Among the 109 males,12(11.00%) were PMs and 97(89.00%) were EMs. In the individual subgroup of Gujratis, 4(8.88%) of the total 45 females and 10(12.98%) of the total 77 males were PMs, the % of PMs in the subgroup as a whole being 11.47%. In the Marwadi subgroup, 1(10%) of the total 10 females and 2(6.25%) of the total 32 were PMs, the % of PMs in the subgroup as a whole being 7.14%.

## Discussion and conclusion

Most of the current literature related to pharmacogenetics of CYP2C19 has studied polymorphisms in Caucasians [10,11], African-American [11] and Oriental populations [11], while far less is known about other ethnic groups. This is the first study from the country to establish the CYP2C19 phenotype frequencies in the Gujrati and Marwadi (Western Indian) population. Indian population comprises of more than 1 billion people. The population is divided into 4 major morphological types – Caucasoid, Mongoloid, Australoid and Negrito. The states of Maharashtra (Maharashtrians), Gujrat (Gujratis) and Rajasthan (Marwadis) belong to the Caucasoid morphological type [21]. All the subjects of the study are actually migrants to the city of Mumbai (Bombay). There were 122 Gujratis and 42 Marwadis. The states of Gujrat and Rajasthan share a common boundary and have socio cultural, and linguistic similarities and hence the total sample size was taken as 164 subjects.

It was seen that omeprazole metabolic ratio is a safe and convenient means for assessing the *in-vivo *activity of CYP2C19. This is in agreement with the use of omeprazole as a probe drug in other ethnic populations [22,23]. The prevalence of PMs in Gujratis and Marwadis was 10.36% by phenotyping, which is slightly lower than frequencies of South Indians (14%) [18], North Indians (12%) [17], Caucasians (3–5%) [10], Africans (8%) [12] and Orientals (12–23%) [11]. There were differences in the distribution of PMs in Gujrati men (12.98%PMs) and women (8.8%PMs);as well as Marwadi men(6.25%PMs) and women(10%PMs), even though the overall incidence of PMs in the study group in both sexes was similar. The number of PMs was too small to do a meaningful statistical analysis. A total of 7/164 subjects were non vegetarians. Of these, 3 were Marwadis and 4 were Gujratis. None of the 7 were poor metabolizers and thus cannot explain the PM status. A total of 10 subjects had a very low MR. It has been reported in literature that super rapid metabolizers exist and these set of patients are non responders to proton pump inhibitors. It has been hypothesized to be caused by variant of the wild type allele. It is possible that these 10 subjects could belong to this category. [24]

CYP2C19 metabolizes many clinically important drugs, notably proton-pump inhibitors, proguanil and benzodiazepines. The clinical implications of the CYP2C19 metabolism are many and varied, ranging from marked efficacy in the treatment of *Helicobacter pylori *infection with omeprazole in PMs to increased risk of failure of anti-malarial prophylaxis with proguanil in the same subgroup. This study was done as a part of a project to determine the polymorphisms of CYP2C19 by phenotyping as well as genotyping. The fact that 10.36% of our study population possesses the PM phenotype makes it essential to evaluate the phenotype-genotypes correlation in this population which can be done after the genotyping data is available. This is required to circumvent adverse drug reactions associated with increased accumulation of the parent drug and would also serve to decrease cost and duration of therapy, as a lower dose may suffice in the individuals who do not metabolize the drug extensively.

## Competing interests

The author(s) declare that they have no competing interests.

## Authors' contributions

TSP and SFN were the undergraduate students who executed the study. They recruited subjects, obtained informed consent, supervised drug administration and collected the blood samples. They also assisted in the statistical analysis. TSP wrote the first draft of the manuscript. SD carried out the entire statistical analysis and contributed to finalizing the methods and statistical section of the manuscript. VMS was responsible for the HPLC analysis. NJG and NAK conceived the study, wrote the protocol, submitted and obtained ethics committee approval, acted as overall project supervisors and made critical contributions to the manuscript at each stage and finalized the paper for submission.

**Table 1:** Data on PMs and EMs in Gujratis and Marwadis

		**Gujratis n = 122**	**Marwadis n = 42**	**Total N = 164**
**Males n = 109**	**PM n = 12**	10 (12.98%)	2 (06.25%)	12 (11.00%)
	**EM n = 97**	67 (87.02%)	30 (93.75%)	97 (89%)
	**total**	77	32	109

**Females n = 55**	**PM n = 5**	4 (08.88%)	1 (10.00%)	5 (09.09%)
	**EM n = 50**	41 (91.12%)	9 (90.00%)	50 (91.91%)
	**total**	45	10	55

## Pre-publication history

The pre-publication history for this paper can be accessed here:


